# Identification of a Metabolic Reprogramming-Associated Risk Model Related to Prognosis, Immune Microenvironment, and Immunotherapy of Stomach Adenocarcinoma

**DOI:** 10.1155/2022/7248572

**Published:** 2022-09-21

**Authors:** Yan Zhao, Dongsheng Zhang, Yueming Sun

**Affiliations:** ^1^Department of Gastrointestinal Surgery, Affiliated Hospital of Jiangnan University, Wuxi, China; ^2^Department of General Surgery, First Affiliated Hospital of Nanjing Medical University, Nanjing, China

## Abstract

Stomach adenocarcinoma (STAD) is one of the most common malignant digestive tumors. Metabolic reprogramming is an essential feature of tumorigenesis. The roles of metabolic reprogramming in STAD patients were investigated to explore the tumor immune microenvironment (TME) and potential therapeutic strategies. STAD samples' transcriptomic and clinical data were collected from The *Cancer* Genome Atlas (TCGA) set and the GSE84437 set. The signature based on the metabolism-related genes (MRGs) was built using the Cox regression model to predict prognosis in STAD. Notably, this MRG-based signature (MRGS) accurately predicted STAD patients' clinical survival in multiple datasets and could serve as an indicator independently. STAD patients with high scores on the MRGS were eligible for generating a type I/II interferon (IFN) response, according to a complete examination of the link between the MRGS and TME. Tumor Immune Dysfunction and Exclusion (TIDE) and immunophenoscore (IPS) analyses revealed that STAD patients with different MRGS scores had different reactions to immunotherapy. Consequently, assessing the pattern of these MRGs increases the understanding of TME features in STAD, hence directing the development of successful immunotherapy regimens.

## 1. Introduction

Stomach adenocarcinoma (STAD) is among the most common digestive malignant tumors. In 2018, approximately one million new cases were reported worldwide, the bulk of which was identified at an advanced stage locally [1, 2]. The prevalence and development of STAD continue to be poorly understood. Existing treatments for STAD mainly include surgery and chemotherapy. After surgery, the rate of local recurrence or distant metastasis varies from 40 to 70 percent, and the adverse effects of radiation and chemotherapy are quickly visible [3]. Consequently, the prevention of STAD has become a pressing public health concern. It is vital to explore the underlying mechanism of STAD to discover novel therapeutic and diagnostic targets that might help to raise the patient survival rate.

Cancers are characterized by metabolic reprogramming, which may contribute to carcinogenesis [4–6]. A large number of studies have pointed out that metabolic phenotypes can be used to image tumors and offer prognostic information, as well as treat malignancies [7]. Targeting certain metabolic pathways as a therapy technique may be beneficial in cancers. For instance, 5-fluorouracil (5-FU) possesses anticancer properties [8]. Previous studies have revealed that the progression of STAD is strongly associated with many different metabolic pathways [9, 10]. In addition, energy metabolism could be a therapeutic focus for STAD patients in the clinic. Nonetheless, the expression patterns of metabolism-related genes (MRGs) involved in metabolic reprogramming remain unclear, as well as their clinical values in STAD. Consequently, systematically evaluating the expression features and clinical importance of those MRGs may be essential for the treatment of patients with STAD.

In this investigation, an MRG-based signature (MRGS) was generated and adequately confirmed by evaluating the transcriptome and clinical data of STAD samples in depth. This research next investigated the connection between the MRGS and other clinicopathologic variables and developed a predictive nomogram. Intriguingly, subsequent investigation revealed that MRGS was strongly linked to immune-related pathways. Consequently, we investigated the associations between the MRGS and tumor immune microenvironment (TME), checkpoint genes, as well as response to immunotherapy and sensitivity to chemotherapeutic treatment.

## 2. Methods

### 2.1. Data Collection

The STAD cohort from The *Cancer* Genome Atlas (TCGA) data portal containing 350 samples and the GSE84437 cohort containing 433 samples were selected for collecting information on STAD samples. Thereafter, the whole TCGA-STAD set was subdivided into a training set and an internal testing set in random order. Besides, we used the whole TCGA-STAD set as another internal validation set, and the GSE84437 set as an external validation set. A total of 1916 specific MRGs that are involved in all the metabolism-associated pathways were downloaded from the c2.cp.kegg.v7.2.symbols.gmt at the GSEA website [11], as shown in Table S1. Besides, data from immunotherapeutic cohorts were obtained from the IMvigor210 (http://research-pub.Gene.com/IMvigor210CoreBiologies) [12].

### 2.2. Identification of Candidate MRGs and Construction of the MRGS

Differentially expressed MRGs were identified between STAD and noncarcinoma samples from the entire TCGA set by using the “limma” package [13]. Then, the candidate MRGs were subsequently extracted from all the differentially expressed ones. The associations of candidate MRGs with the overall survival (OS) of STAD patients from the training set were analyzed using univariate Cox regression. The most optimal genes were selected via using the LASSO regression through a package named “glmnet” [14]. Thereafter, the multivariate Cox regression based on the optimal genes was used for confirming hub genes to construct the MRGS. Based on the median one of all MRGS scores in the training set, STAD patients in all sets were separately subdivided into the low- or high-risk group.

### 2.3. Evaluation of the Constructed Model's and Nomogram's Prognostic Value

The “survival” *R* package plotted the Kaplan–Meier analysis of all STAD groups [15]. In addition, the plotted ROC curves were to determine the signature's specificity, as well as its sensitivity [16]. The entire TCGA cohort was utilized for analyzing the independence of the MRGS along with several common clinical variables. Combining these clinical variables with the constructed MRGS, we built a prognostic nomogram to help to assess the survival probability of STAD patients quantitatively [17].

### 2.4. Analysis of Immune Cell Infiltration Level and Enriched Pathways

To analyze the correlation between the built MRGS and immune cell infiltration, we estimated the 22 immune cell subtype infiltration levels by CIBERSORT [18]. GSVA analysis was carried out on the gene expression through the package named “GSVA” to explore the biological process distinction.

### 2.5. Immunotherapy Efficacy Based on the MRGS

The tumor mutation burden (TMB) was calculated for each sample from the entire TCGA set. The checkpoint gene level was analyzed for confirming their relationship with the clinical OS of patients with STAD. Tumor Immune Dysfunction and Exclusion (TIDE, http://tide.dfci.harvard.edu/) [19] is designed to examine immune evasion mechanisms. It serves as an additional reliable biomarker that is usually used to predict immunotherapy efficacy. Greater TIDE scores suggest that tumor cells are more likely to elude immunosurveillance, hence implying a lower rate of immunotherapy response. The immunophenogram (IPS) of The *Cancer* Immunome Atlas (TCIA, https://home.at/) database was also used to assess the response of STAD patients to immune checkpoint inhibitors (ICIs) [20]. A higher IPS score frequently implies a more favorable immunotherapy response.

### 2.6. Chemotherapy Sensitivity Analysis

CellMiner [21] (https://discover.nci.nih.gov/cellminer) was used to access the NCI-60 database, which comprises a total of 60 cancer cell lines derived from various kinds of malignancies. We carried out Pearson correlation analyses to determine the relationship between the MRGS values and sensitivity to chemotherapeutic drugs.

### 2.7. Statistical Analysis

In this study, statistical analysis was carried out using the SPSS and *R* software (version 3.5.1). The “survival” package was used for the Kaplan–Meier analyses, as well as the univariate and multivariate Cox regression analyses. And, the risk ratios and accompanying confidence intervals of 95 percent were gathered. For the in vitro experiments, independent sample *t*-tests were performed for the comparisons between groups. *P* values less than 0.05 were considered statistically significant.

## 3. Results

### 3.1. Identification of Hub MRGs and Construction of the MRGS

**Figure 1:**
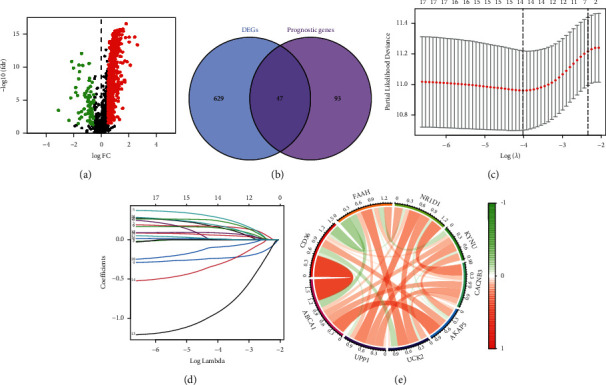
Establishment of MRGS. (a) Volcano plot regarding MRGS that differentially expressed between STAD samples and normal samples. (b) The intersections of the differentially expressed MRGS and the MRGS with prognostic value for STAD. (c) LASSO Cox regression analysis for STAD samples based on the MRGS in the intersections. (d) Coefficient profiles from the LASSO Cox analysis. (e) Correlation network of the nine candidates MRGS.

**Figure 2:**
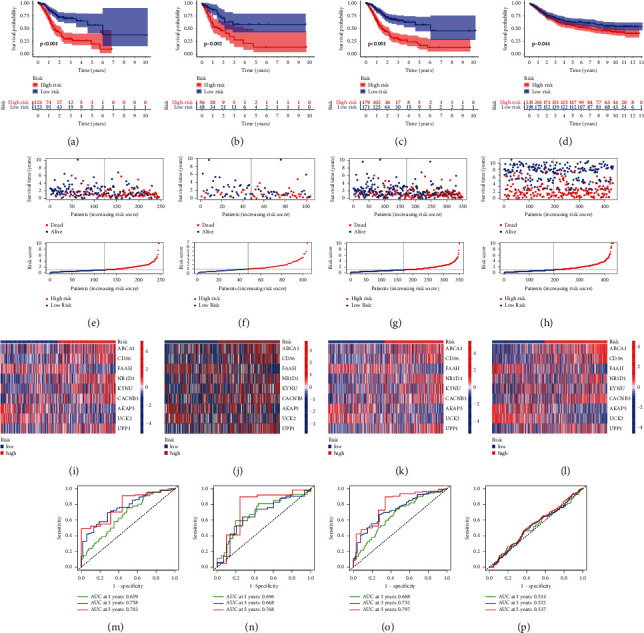
Evaluation of MRGS in predicting the survival of STAD samples from different cohorts. Distribution of KM survival (a-d), risk scores and survival status (e-h), hub MRGs' expression levels in different STAD groups (i-l), and time-dependent ROC analyses (m-p) on the TCGA training set (a, e, I, and m), TCGA testing set (b, f, j, and n), entire TCGA cohort (c, g, k, and o), and GSE84437 cohort (d, h, l, and p).

By comparing the genes' expression levels in the STAD and normal samples, we obtained 676 differentially expressed MRGs (Figure 1(a)). Meanwhile, we acquired 140 MRGs associated with the OS of STAD samples via the univariate analysis. Then, a total of 47 MRGs were extracted (Figure 1(b)). To explore the MRGs that were closely related to the STAD prognosis in the clinic, we carried out a univariate analysis based on the 47 chosen genes and found 18 related genes. The most appropriate tuning parameter from the LASSO Cox analysis was chosen later for preventing the overfitting based on the 18 ones (Figures 2(c) and 2(d)). Finally, a total of 9 MRGs were selected as hub ones, including ABCA1, CD36, FAAH, NR1D1, KYNU, CACNB3, AKAP5, UCK2, and UPP1. Afterward, the MRGS was established according to the expression of these 9 hub MRGs along with their multivariate Cox regression coefficients. The formula is as followed: score = (0.2684 × ABCA1 level) + (0.2350 × CD36 level) + (−0.3043 × FAAH level) + (0.3981 × NR1D1 level) + (0.1651 × KYNU level) + (0.2927 × CACNB3 level) + (−1.2282 × AKAP5 level) + (-0.5845 × UCK2 level) + (0.2684 × UPP1 level). Moreover, the 9 prognostic MRGs were correlated with each other (Figure 1(e)).

### 3.2. Valuation of the Predicting Ability of  MRGS

Based on the MRGS, we calculated each STAD sample's score and divided all STAD samples into the low-risk group or the high-risk group (Figure 2). Figures 2(a)–2(d) show that STAD samples in the low-risk group owned favorable survival when they were compared with those in the high-risk group in multiple sets. The risk scores and survival status of STAD samples from the multiple sets are shown in Figures 2(e)–2(h). The expression of hub MRGs in the proposed signature was similar in multiple sets (Figures 2(i)–2(l)). Furthermore, ROC analyses were carried out to evaluate the risk model's prediction (Figures 2(m)–2(p)). The values of the area under the ROC curves performed in the TCGA training set were 0.659, 0.758, and 0.783, separately for 1-, 3-, and 5- year survival, suggesting that MRGS had a good performance in monitoring survival. Meanwhile, MRGS had highly accurate predictions for the survival of STAD samples in the TCGA testing set, the whole TCGA set, and the GSE84437 set.

### 3.3. Association of the MRGS and Clinical Variables in STAD

**Figure 3:**
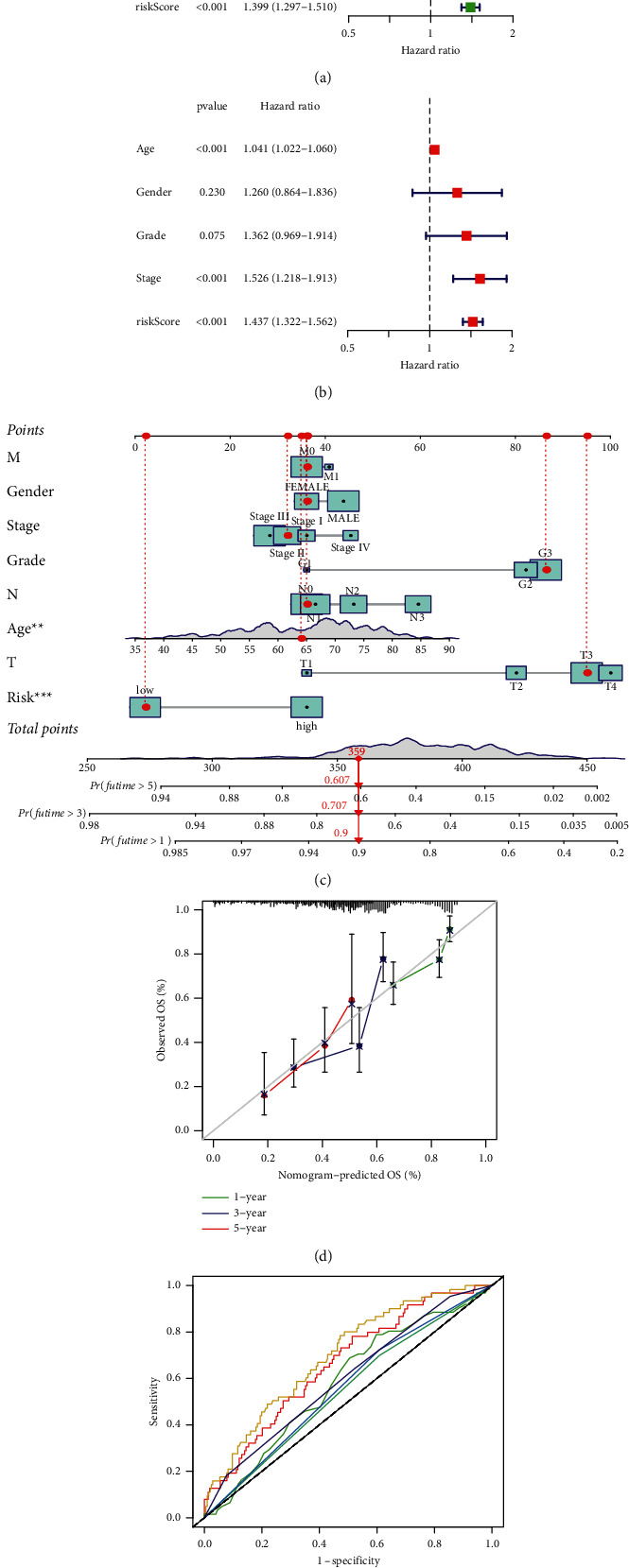
The dependence of the MRGS for prognostic prediction in STAD. (a) Results of the univariate Cox analyses of the MRGS and multiple clinical features in patients from the entire TCGA-STAD set. (b) Results of the further multivariate Cox analyses. (c) Nomogram predicting the OS in the entire TCGA cohort. (d) Calibration curves of nomogram on the consistency. (e) ROC analysis of the constructed clinical nomogram by comparing it with other chosen clinical variables.

We carried out the univariate and multivariate Cox regression analyses on the MRGS and common clinical features in the entire TCGA-STAD cohort. Figures 3(a) and 3(b) demonstrate that the MRGS was significantly associated with the OS of STAD patients, suggesting that the generated MRGS may serve as a factor independently predicting the clinical prognosis. Noteworthily, age and tumor stage were also significantly correlated with the OS (Figures 3(a) and 3(b)). In addition, we developed a clinical nomogram based on the MRGS and multiple chosen clinical factors to objectively estimate the survival likelihood of individuals (Figure 3(c)). In general, the calibration curves of our generated prognostic nomogram were very congruent with the anticipated and observed survival rate in the whole TCGA cohort (Figure 3(d)). In addition, the AUC of the nomogram was greater than that of other clinical variables in the ROC curve (Figure 3(e)), demonstrating the nomogram's superior performance.

**Figure 4:**
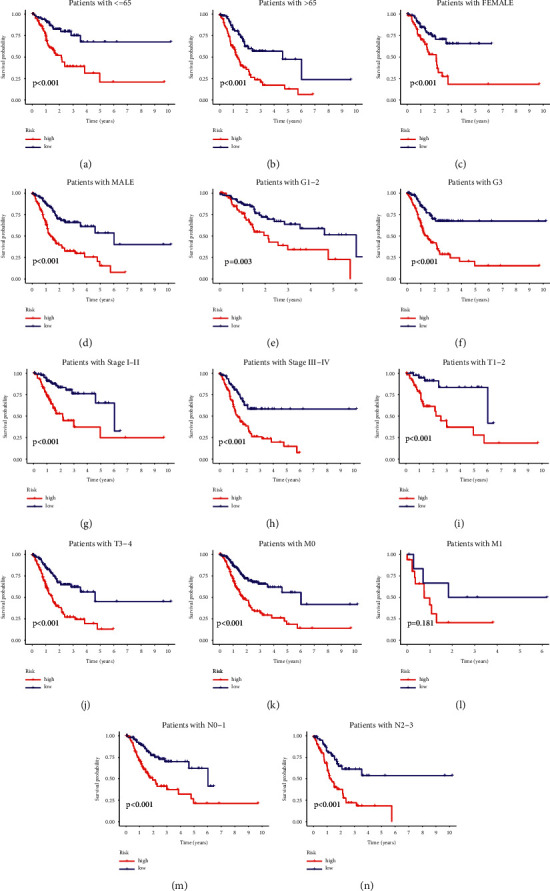
Stratified analysis based on the built model and clinical stratifications. (a-m) Longer survival time was obviously observed in STAD patients with low scores in most clinical stratifications, including patients' age (a and b), patients' gender (c and d), tumor grade (e and f), tumor stage (g and h), tumor T stage (I and j), tumor M stage (k and l) and tumor N stage (m and n).

Subgroup analysis was carried out to see whether the built signature still had independent predictive value for the most important clinical characteristics.  Figure 4 demonstrate that the MRGS retained its predictive power in subgroups defined by age (age > 65 or ≤ 65, Figures 4(a) and 4(b)), gender (female or male, Figures 4(c) and 4(d)), tumor grade (G1-2 + G3, Figures 4(e) and 4(f)), tumor stage (SI-II + SIII-IV, Figures 4(g) and 4(h)), *T* stage (T1-2 + T3-4, Figures 4(i) and 4(j)), *M* stage (*M*0 + M1, Figures 4(k) and 4(l)), and N stage (N0-1 + N2-3, Figures 4(m) and 4(n)). In subgroups with distinct clinical features, the OS duration of low-risk samples was manifestly longer than that of high-risk samples.

### 3.4. Interrelation of the  MRGS, Immune Cell Infiltration, TME, and TMB

**Figure 5:**
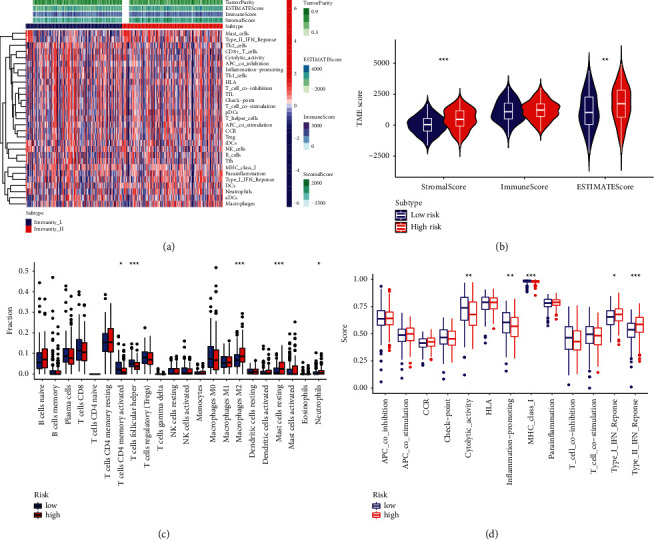
Correlation between MRGS and immune cell infiltration, TME. (a) Correlation between the MRGS and immune cell infiltration. (b) Results of ssGSEA analysis on the immune-related functions between two risk groups. (c) The landscape of the immune characteristics and TME. (d) Correlations between MRGS score and TME score.

To completely characterize the immunological aspects of STAD, CIBERSORT was carried out to examine the infiltration of immune cell subtypes in the whole TCGA-STAD set. The relative abundance of activated memory CD4 T cells was significantly negatively related to the score, and so did that of the follicular helper T cells (Figure 5(a)), whereas the relative abundance of M2 macrophages and resting mast cells were significantly positively correlated with the score (Figure 5(a)). In addition, type I and type II interferon (IFN) responses were both activated in the group with high risks, suggesting that immunosuppressed STAD patients might react to immunotherapy (Figure 5(b)). To further investigate the biological behaviors, a GSVA enrichment analysis was undertaken. Interestingly, many metabolism pathways, including selenoamino acid metabolism, glyoxylate and dicarboxylate metabolism, and cysteine and methionine metabolism, were significantly enriched in the low score group (Figure S1).

**Figure 6:**
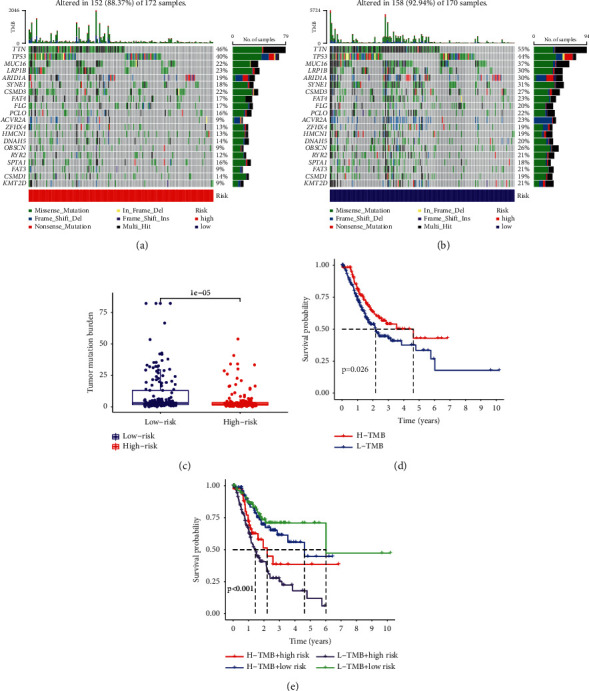
Estimation of TME and TMB in the entire TCGA-STAD set. (a) and (b) Genes with high mutation frequencies in different risk STAD groups. (c) Difference of TMB between two STAD groups. (d) Kaplan–Meier analysis based on the TMB. (e) Kaplan–Meier analysis for four groups that were stratified by combining the constructed MRGS and the TMB.

On the basis of MRGS scores and the hierarchical clustering algorithm, all STAD samples from the entire TCGA set were neatly divided into two groups (Figure 5(c)). The features of the TME between the two STAD groups were discovered based on the findings of ESTIMATE. We discovered that the groups with the higher MRGS scores had higher estimate score and stromal score levels than the other group, which had lower values (Figure 5(d)). The mutation data were examined using the maftool package, and the mutations were stratified according to the variant effect predictor. Figures 6(a) and 6(b) depict the top 20 driver genes with the greatest frequency of modification between the high- and low-risk STAD groups. The difference in TMB between groups was also shown to be statistically significant (Figure 6(c)). Clearly, a high TMB was connected with a healthy clinical OS (Figure 6(d)). We investigated if the combination of the MRGS and TMB may be a more accurate prognostic biomarker. Therefore, we used MRGS and TMB to stratify all STAD samples from the entire TCGA set into four distinct groups. As seen in Figure 6(e), there were substantial disparities between all four groups. Moreover, the individuals with the highest TMB and lowest MRGS scores had the greatest OS. These findings indicated conclusively that MRGS was positively associated with tumor malignancy.

### 3.5. Correlation of Checkpoint Genes and the MRGS and Their Impact on Clinical Outcome in the Entire TCGA-STAD Cohort

**Figure 7:**
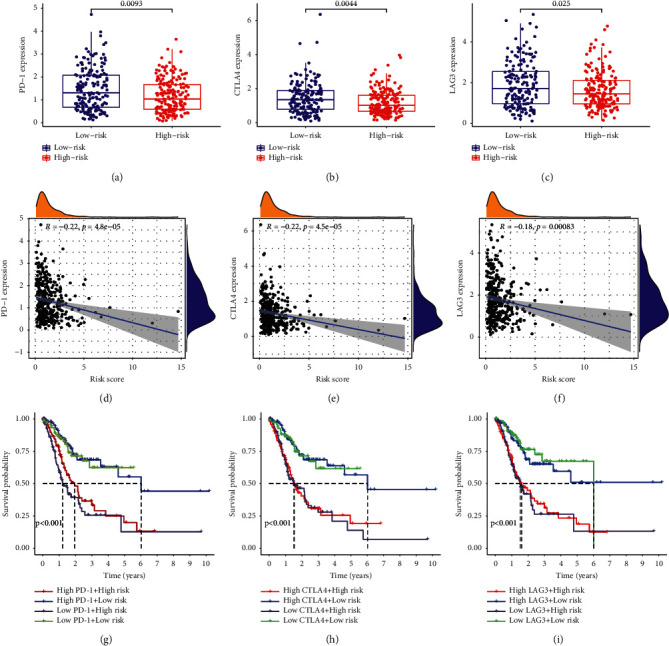
Correlation of checkpoint genes and the MRGS and their impact on clinical outcome. (a-c) Comparison of the PD-1, CTLA-4, or LAG3 expression levels between groups with different risks. (d-f) Correlation between the MRGS score and checkpoint gene (PD-1, CTLA-4, or LAG3) expression level. (g-i) Kaplan–Meier analyses the clinical OS in the four groups grouped by the MRGS and the level of PD-1, CTLA-4, or LAG3.

Previous research has shown the significance of immune checkpoint genes in regulating immune infiltration [22–24]. To further study the complicated interplay between immune checkpoints and the established MRGS, we evaluated their expression patterns across MRGS-based groups. As shown in Figures 7(a)–7(c), STAD patients with higher MRGS scores expressed lower levels of three chosen immune checkpoint genes (PD-1, CTLA4, and LAG3) in the entire TCGA set. Meanwhile, the expression levels of three chosen checkpoint genes all showed negative correlations to the MRGS scores (Figures 7(d)–7(f)). Then, we analyzed MRGS in conjunction with immune checkpoint expression to determine if MRGS affects the OS of STAD patients with comparable checkpoint genes' expression. Survival analysis was carried out on four groups that were stratified by MRGS and immune checkpoint gene expression.  Figure 7(g) illustrate that those individuals with higher PD-1 expression levels and lower MRGS scores had a longer OS than those with higher PD-1 expression levels and higher MRGS scores. In individuals with low PD-1 expression levels, a lower risk score indicated a survival rate that was significantly improved. In the entire TCGA-STAD cohort, similar survival trends were identified across the four STAD patient groups stratified by the MRGS scores and CTLA4 (Figure 7(h)) or LAG3 (Figure 7(i)) expression.

### 3.6. Predictive Potential of the MRGS in Immunotherapy Response and Drug Sensitivity

**Figure 8:**
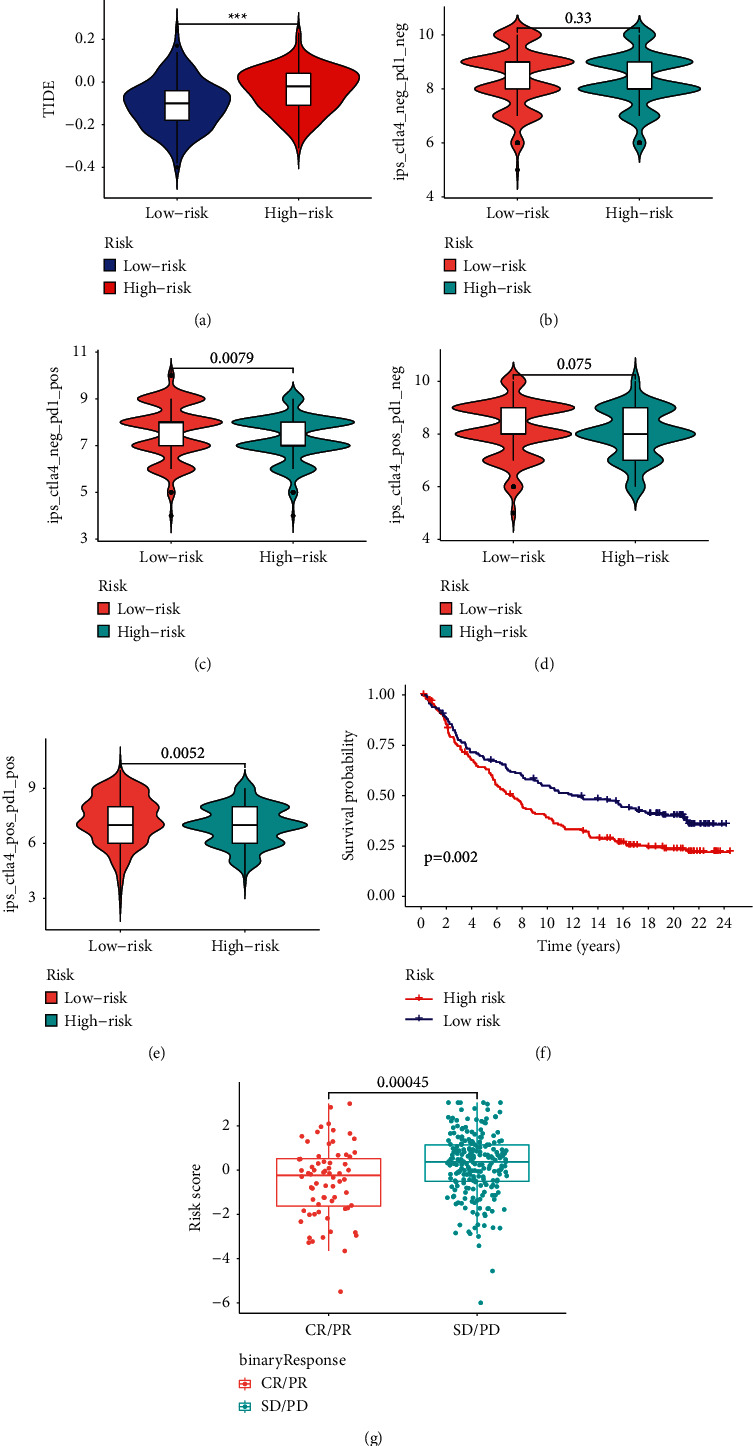
Predictive potential of the MRGS in immunotherapeutic benefits. (a) The TIDE scores between STAD patients with different MRGS scores. (b-e) The association between the MRGS score and IPS. (f) Kaplan–Meier analysis based on the IMvigor210 cohort. (g) The distribution of MRGS in the binary response.

There is mounting evidence that ICIs increase STAD survival, although responses vary. Therefore, precise prognostic biomarkers are urgently required. In light of the link between the MRGS and immune infiltration, as well as the checkpoint gene levels, we investigated the predictive ability of MRGS by analyzing its correlation with known immunotherapy predictors, such as TIDE [25, 26] and IPS [27]. High-risk STAD patients tended to attain greater TIDE scores in the TCGA cohort, suggesting that those in the group with low scores may benefit from ICIs in the clinic (Figure 8(a)). IPS serves as a superior predictor for the response to anti-CTLA-4 antibodies and anti-PD-1 antibodies. Although our results showed that there was no difference in IPS between the two groups shown in Figures 8(b) and 8(d), the IPS scores in the low-risk STAD group in Figures 8(c) and 8(e) were significantly elevated, suggesting that these patients may have better responses to ICIs. In addition, given the immunotherapy response prediction capacity of the MRGS, we ran Kaplan–Meier analyses on the immunotherapy cohort (IMvigor210) to evaluate the predictive significance of the immunotherapeutic OS. The anti-PD-L1 clinical response in the IMvigor210 cohort was classified as partial response (PR), complete response (CR), progressing disease (PD), and stable disease (SD). As anticipated, low scores were found to have a favorable trend in the immunotherapeutic OS (Figure 8(f)). The MRGS also had meaningful differences between the CR/PR and SD/PD groups from the IMvigor210 cohort (Figure 8(g)). All the results above showed that the constructed MRGS performs well in predicting the response to immunotherapy for STAD patients.

**Figure 9:**
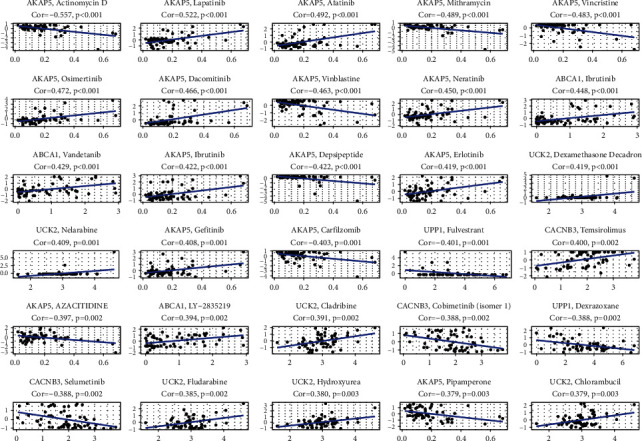
The relationship between MRGs' expression and drug sensitivity.

Moreover, we investigated the expression of prognostic MRGs in the proposed signature in cancer cells from the NCI-60 database. The results in Figure 9 showed that the majority of these prognostic MRGs were closely associated with sensitivity to some chemotherapy drugs. For example, increased expression of AKAP5, ABCA1, UCK2, and CACNB3 was obviously related to the increased drug resistance to lapatinib, afatinib, osimertinib, dacomitinib, neratinib, ibrutinib, etc. On the contrary, elevated expression levels of UPP1, CACNB3, and AKAP5 were significantly related to the increased drug sensitivity to fulvestrant, dexrazoxane, actinomycin *D*, selumetinib, pipamperone, etc.

## 4. Discussion

Extensive research shows that cells in cancers usually exhibit abnormal metabolic characteristics [28]. Metabolic reprogramming is a crucial characteristic of cancer genesis. Changes in cellular metabolic activity are a characteristic of cancer [29]. One of the physiological hallmarks of a human malignant tumor is an elevated glycolytic metabolism, for instance [30]. Several studies have shown that metabolic markers like cysteine metabolism, nucleotide metabolism, and 2-hydroxyglutarate may be used to categorize and treat gliomas [31, 32]. Given the above, metabolic therapy is a viable therapeutic option for STAD.

Here, we analyzed the MRGs' levels, together with their prognostic value based on the transcriptomic data of patients with STAD. As a result, 9 identified MRGs were adopted for building the MRGS. The mRNA level of NR1D1 was upregulated in STAD tissues when compared with normal tissues [33]. The prognosis of STAD patients likely benefited from lower expression levels of KYNU [34]. Low expression of AKAP5 may be a potential molecular marker for predicting poor prognosis of non-mucin-producing stomach adenocarcinoma (NMSA) via regulating cholesterol homeostasis, estrogen response, glycolysis, notch signaling, and adipogenesis pathways [35]. The malignant cell marker, UPP1, was selected to generate a signature for STAD patients [36]. Although other MRGs in the proposed signature have not been investigated in STAD previously, they have been confirmed to influence the progression of cancers [37–41]. According to the median MRGS value of all scores, we grouped the STAD samples from different sets into low-risk or high-risk groups. The findings demonstrated that STAD patients with lower model scores had superior survival to those with higher MRGS scores. The ROC curve analysis confirmed that the MRGS had a high potential for predicting survival. Moreover, the univariate or multivariate analysis based on MRGS and other clinical variables confirmed the independence of the MRGS's prognostic value, while the OS time of STAD samples varied significantly in different clinical feature subgroups. The nomogram we built based on the MRGS and common clinical variables could better predict the survival of STAD patients since its AUC value was a little higher.

Numerous landmark studies have pointed out that metabolic changes exert vital roles in immune regulation [42, 43]. The aberrant metabolism in cancers may have a substantial effect on TME, which is often acidic, hypoxic, and lacks nutrients necessary by immunological cells [44]. In addition, aerobic glycolysis inside cancer cells shapes the immune system by upregulating cytokine production and inhibiting the development of monocytes into dendritic cells [45]. Meanwhile, immune cells' metabolism is a significant factor in determining their survivability and roles [46]. Consequently, metabolism is strongly tied to immunity. The creation of a prognostic signature related to metabolism may aid a lot in forecasting the status of immune responses. To better understand the relationship between the built MRGS and immunity, we compared TME between groups and found several immune cell infiltration levels were obviously elevated in the STAD group with low risk. These findings indicate that metabolically active tumor cells in STAD formed a microenvironment that was harmful to immune cells, consistent with previous reports [47]. The immune response against cancer is an important component of the complicated tumor immunophenotype that underlies the TME [48]. By modifying the immune cell state inside TME, the aberrant metabolism may contribute to a different outcome. This is consistent with the finding that tailored metabolism may facilitate the regulation of antitumor immune response.

Interestingly, our data showed that the main indicators of exhausted T cells were abnormally elevated in STAD samples with higher MRGS scores, suggesting T cells may have become more hypofunctional and hyporesponsive as metabolism activates in cancer cells. This result may explain why elderly persons have a lower immunotherapy response rate. In order to confirm our findings, we also collected immunotherapy data from TICA. STAD patients with lower MRGS scores may have a stronger immunotherapy response, as evidenced by the findings. Moreover, we collected immunotherapy information from the IMvigor210 set and further confirmed the ability of the MRGS in predicting the STAD patients' immunotherapy responses.

The current work has a number of strengths. This is the first signature that is created on the basis of metabolic reprogramming that may represent the prognosis and TME, immunotherapy of STAD patients. Second, different data sets were used in order to validate and assess the predictive efficacy of our created signature. Thirdly, we determined the possible molecule that matched our signature. However, more prospective cohort studies are required to evaluate the therapeutic utility of this predictive signature. In the meanwhile, the possible chemical requires additional investigation.

## 5. Conclusion

In conclusion, we performed a complete examination of the expression of MRGs in STAD patients and then developed an MRGS with the ability to predict clinical outcomes and immunological microenvironment. As a useful tool, this built signature may aid in searching for possible combination immunotherapy drugs and offer a therapeutic approach for the treatment of STAD patients.

## Data Availability

Publicly available datasets were analyzed in this study. This data can be found in TCGA-STAD and GSE84437.

## Supplementary Materials

Supplementary Materials Figure S1. The GSVA enrichment heatmap between two risk score groups from the entire TCGA-STAD set. Table S1. The details of MRGs.()
