# Supplementary material for: Identification of a Metabolic Reprogramming-Associated Risk Model Related to Prognosis, Immune Microenvironment, and Immunotherapy of Stomach Adenocarcinoma
Source: J Oncol. 2022 Sep 21;2022:7248572. doi: 10.1155/2022/7248572 (PMC9519326; doi:10.1155/2022/7248572)
Supplement: Supplementary Materials — Figure S1. The GSVA enrichment heatmap between two risk score groups from the entire TCGA-STAD set. Table S1. The details of MRGs.() [file 7248572.f1.zip › supplementary table 1.docx]

| Gene Name |
| --- |
| HSD3B1  HSD3B2  AP2A1  AP2A2  AP2B1  AP2M1  A2M  AP2S1  PRKAA2  PRKAB2  PRKAG2  ABCB11  ABCA1  ABCG5  ABCG8  DBI  ACADL  ACADM  ACADS  ACADVL  ACHE  ACLY  SCD  NDUFAB1  AGPS  PLIN2  FDXR  FDX1  GLA  AHR  AKR1C1  AKR1C2  AKR1C3  AKR1C4  AKR1D1  ALB  AKR1B1  ABCD1  AMACR  AGT  ANGPTL3  ANGPTL4  APOA1  APOA2  APOA4  APOB  APOC1  APOC2  APOC3  APOC4  APOE  APOF  ACER3  LPA  ARF1  ARF3  ARNT2  ARNT  ARSA  ARSB  ARSD  ARSE  ARSF  ASAH1  SMPD1  ACOT7  CEL  BDH1  GLB1  BHMT  BMX  CYP11A1  CYP39A1  ACOT12  CRAT  B4GALNT1  ACOX1  ACOX2  ACOX3  CAV1  NFYB  CREBBP  CD36  CDS1  CDS2  UGCG  CETP  ACOT9  CCNC  UGT8  BCHE  CIDEA  CLOCK  CPNE1  CPNE3  CPNE6  CPNE7  ACACB  POMC  CLPS  COQ3  COQ6  COQ7  CYP1A1  CYP1A2  CYP1B1  CYP27A1  CYP46A1  CYP4B1  CYP4A11  CYP51A1  CYP7A1  CYP7B1  CYP8B1  CYP2C8  CYP2C9  CYP2C19  CYP4F2  CYP4F3  CYP4F11  CYP2J2  CYP11B1  CYP11B2  CYP21A2  CPT1A  CPT2  CYP17A1  CPT1B  CYP19A1  MED17  MED7  SPTLC3  FITM2  CTGF  PCYT1A  PCYT1B  MID1IP1  ALDH7A1  DAB1  GNPAT  MLYCD  DECR1  DGAT1  ALDH3B1  ALDH3B2  HSD17B1  HSD17B2  HSD17B3  HSD17B4  HSD17B8  DHCR7  CBR1  DHCR24  HSD11B1  HSD11B2  EBP  HADHA  HADHB  ECHS1  EHHADH  ETNK1  ETNK2  ELOVL1  ELOVL2  ELOVL3  ELOVL4  MVD  TM7SF2  SQLE  LSS  ESRRA  FAAH  FABP4  FABP7  FABP5  FABP3  FABP2  FABP1  FASN  FDFT1  FGF21  ALOX5AP  FDPS  CIDEC  FURIN  KDSR  FYN  PIKFYVE  G0S2  GALC  GGPS1  GGT1  GGT5  GK2  GBA  CGA  GLIPR1  GK  GLTP  GPD1  GPD2  PNPLA4  GPX1  GPX2  GPX4  TECR  HAO2  HADH  ALAS1  HEXA  HEXB  HMGCS2  HMGCS1  HACL1  HRASLS5  HRASLS  HRASLS2  PLA2G16  EPHX2  IDH1  IDI1  IDI2  FABP6  KPNB1  INSIG1  PRKACA  PRKACB  PRKACG  CSNK1G2  CSNK2A2  CSNK2B  GPCPD1  CHKB  CHKA  MVK  PRKD1  PRKD3  PRKD2  PTGR1  LTC4S  LCAT  SPTLC1  SPTLC2  ACSL1  ACSL3  ACSL4  ACSL5  ACSL6  LDLR  VLDLR  LIPA  PNLIPRP1  LIPG  LIPF  LIPC  LPL  PNLIP  LIPE  LTA4H  ALOX15  ALOX5  ALOX12  ALOX12B  LPIN1  LPIN2  LPIN3  LHB  ALOX15B  DMGDH  ME1  SLC25A20  DPEP1  ABCB4  MED6  STARD3NL  MAPKAPK2  STARD3  ABCC1  ABCC3  MBTPS1  MBTPS2  MTM1  MTTP  MTMR1  MTMR2  MTMR3  MTMR6  MTMR7  MUT  NCOA2  NEU1  NEU2  NEU3  TRIB3  SCP2  NPAS2  NPC1  NPC2  NR1D1  NRF1  NR1H2  NSDHL  SMPD2  SLC10A2  SLC10A1  CROT  OSBPL1A  OSBPL2  OSBPL3  OSBPL5  OSBPL6  OSBPL7  OSBPL8  OSBPL9  OSBPL10  OSBP  PIK3CA  PIK3CB  PIK3CD  PIK3CG  EP300  PIK3R3  PIK3R1  PIK3R2  PLA2G1B  PLA2G3  PLA2G4A  PLA2G5  PLA2G6  PLA2G2A  PLA2G2D  PLA2G2E  PLA2G2F  PLA2G10  PLA2G12A  PHYH  PCCA  PCCB  PCSK5  STARD10  PCYT2  P4HB  PEMT  HPGDS  HPGD  PTGS1  PTGS2  PTGDS  PI4KA  PIP4K2A  CDIPT  PIK3C2B  PIK3C2G  AGPAT1  AGPAT2  AGPAT3  AGPAT4  AGPAT5  PLD2  PLIN1  GPAM  PLTP  SLC25A17  PMVK  ACOT13  PON1  PON2  PON3  PPP1CA  PPP1CB  PPP1CC  PPARA  PPARD  ALPI  PCTP  PITPNB  PPT1  PPT2  CTSA  PTDSS1  ACOT8  ACOT2  PTEN  PTGES  PTGIS  PTPN13  RAB14  RAB5A  RELN  RGL1  RORA  RXRA  SLCO1A2  SLCO1B1  SLCO1B3  SEC23A  SEC24A  SEC24B  SEC24C  SEC24D  SRD5A1  GM2A  PSAP  SAR1B  SCAP  OXCT1  FHL2  SOAT1  SOAT2  SP1  SPHK1  SPHK2  MED21  SREBF2  STAR  STARD4  STARD5  STARD6  STARD7  STS  SULT2A1  MED22  SYNJ1  SYNJ2  MED24  THRAP3  MED12  MED13  TBL1XR1  TBL1X  TEAD1  TEAD2  TEAD3  TEAD4  PTGES3  TAZ  TBXAS1  ACOT11  THRSP  ACAA1  ACAT1  ACAA2  PLIN3  RARRES3  TPTE  TNFRSF21  TXNRD1  SLC25A1  VAPA  VAPB  HDLBP  SLC27A2  LPGAT1  FIG4  YAP1  ZDHHC8  CDK8  RAN  RAB4A  PLD1  LBR  MCEE  HMGCL  PNPLA3  AMN  CERK  TMEM55B  CRLS1  DDHD1  MMAA  PI4KB  INPP5J  PCSK9  EPT1  SGPL1  SH3KBP1  INPP5K  SIN3A  SIN3B  MED31  SGPP1  SGPP2  SCARB1  SUMF1  SUMF2  MED20  HSD3B7  B3GALNT1  ARNTL  BMP1  COL4A3BP  CHDH  ACOT1  ALDH3A2  MCAT  HMGCR  INPP5E  MGLL  MYLIP  PLA2G4C  PIP4K2B  PLEKHA1  PLEKHA2  PLEKHA3  PLEKHA4  PLEKHA5  PLEKHA6  PPARGC1A  PTDSS2  ACOT4  RUFY1  SARDH  OLAH  SGMS1  SGMS2  SREBF1  ACAT2  BAAT  THEM4  LDLRAP1  APOA5  PIK3C3  PIK3R4  CH25H  SLC27A5  TGS1  CSNK2A1  CYP4F22  MED25  MED14  MED30  MED4  AACS  ABHD3  ABHD4  ABHD5  ACBD4  ACBD5  ACBD6  ACBD7  ACSBG1  ACSBG2  ACAD10  ACAD11  ACOT6  ACOXL  ACSF2  ACSF3  ACSM3  ACSS3  NCEH1  AGK  ANKRD1  ARSG  ARSH  ARSI  ARSJ  ARSK  ARV1  ACER2  ASAH2  ACER1  AWAT1  AWAT2  BDH2  CARM1  CBR4  CDK19  CEPT1  CHD9  CHPT1  COQ2  COQ5  COQ9  CREB3L3  SLC44A1  SLC44A2  SLC44A3  SLC44A4  SLC44A5  CUBN  DDHD2  DECR2  DEGS1  DEGS2  DGAT2L6  DGAT2  HSD17B11  HSD17B12  HSD17B13  HSD17B14  PDSS2  DPEP2  DPEP3  PDSS1  ELOVL5  ELOVL6  ELOVL7  ENPP6  ENPP7  CES3  ESYT1  ESYT2  ESYT3  FAAH2  FABP9  FAR1  FAR2  FADS1  FADS2  GBA2  GDE1  GDPD1  GDPD3  GDPD5  GLB1L  GLTPD1  GPD1L  GRHL1  GPIHBP1  INPP4A  INPP4B  INSIG2  LIPH  LIPI  LIPJ  LIPK  LIPM  LIPN  PNLIPRP3  LMF1  LMF2  LSR  LCLAT1  MBOAT1  MBOAT2  LPCAT3  MBOAT7  MED13L  MED10  MED11  MED18  MED19  MED28  MED29  MED9  MFSD2A  MOGAT1  MOGAT2  MOGAT3  MTF1  MTMR4  MTMR14  NPC1L1  SMPD3  SMPD4  NUDT19  NUDT7  ORMDL1  ORMDL2  ORMDL3  PIK3C2A  PI4K2A  PI4K2B  PLA2G4D  PLA2G4E  PLA2G4F  LPCAT1  LPCAT2  PECR  PTGES2  PHOSPHO1  PIK3R5  PIK3R6  PIP4K2C  PIP5K1A  PIP5K1B  PIP5K1C  PITPNM1  PITPNM2  PITPNM3  PLEKHA8  PLA1A  PLA2R1  PLB1  PLBD1  AGPAT6  LPCAT4  AGPAT9  PLD3  PLD4  PNPLA2  PNPLA5  PNPLA6  PNPLA7  PNPLA8  GPAT2  ACP6  PPM1L  PPARGC1B  PTPLAD1  PTPLAD2  PTPMT1  PEX11A  SLC27A1  SLC27A3  TECRL  SRD5A3  SACM1L  SCD5  INPP5D  INPPL1  SMARCD3  TNFAIP8  THEM5  TMEM86B  TNFAIP8L1  TNFAIP8L2  TPTE2  FITM1  VAC14  WWTR1  PLD6  PTGR2  CHAT  C10orf129  AHRR  FAM120B  HMGCLL1  PGS1  INPP5F  TNFAIP8L3  NCOA6  CYP4A22  FABP12  PTPLA  PTPLB  HDAC3  NCOR1  MED26  CLTA  NFYA  PCSK6  PPARG  ABCG1  HSD17B7  CLTC  OCRL  ACACA  NR1H3  NFYC  MED1  NCOA1  MED27  CYP2U1  TIAM2  MED8  NR1H4  MED15  MED23  MED16  NCOR2  NCOA3  PISD  NEU4  AKR1B15  PPP2R1A  PPP2R1B  PGD  PGLS  AAAS  GOT1  GOT2  AKR1A1  AKR1B1  ALDOA  ALDOB  ALDOC  AMY2B  AMY2A  AMY1A  NAGLU  ARSB  B3GAT1  B3GAT2  B3GAT3  B3GNT1  B3GNT2  B3GNT3  B4GALT1  B4GALT2  B4GALT3  B4GALT4  B4GALT5  B4GALT6  B4GALT7  GLB1  GUSB  CALM1  CD44  SLC25A12  SLC25A13  CRYL1  DERA  ALDH1A1  SORD  SLC25A10  SLC26A2  ENO1  ENO3  ENO2  EXT1  EXT2  FBP1  FBP2  PFKFB1  PFKFB2  PFKFB3  PFKFB4  FGF21  FMOD  GAPDH  GAPDHS  G6PD  GPI  G6PC  GALNS  GALK1  GALT  GALE  GCKR  AGL  GNS  GLCE  GBE1  GYG1  GNPDA1  GPC1  GPC3  GPC4  GPC5  GPC6  SLC2A1  SLC2A2  SLC2A3  SLC2A4  SLC2A5  GYG2  GYS1  GYS2  HAS1  HAS2  HAS3  HEXA  HEXB  HMMR  NDST1  NDST2  HK1  HK2  HK3  HYAL2  IDS  IDUA  NUP160  PFKM  PFKL  PFKP  PRKACA  PRKACB  PRKACG  KERA  KHK  PHKA1  PHKA2  PHKB  PHKG1  PHKG2  PRPS1  PRPS2  PRPS1L1  PKLR  LALBA  LCT  LUM  GAA  MAN2B1  MAN2B2  MAN2C1  SLC25A11  MANBA  MDH1  MDH2  MGAM  OGN  ABCC5  NUP107  NUP153  NUP214  SLC9A1  NUP37  NUP43  NUP50  NUP62  NUP88  OMD  PPP2CA  PPP2CB  HSPG2  ACAN  NCAN  VCAN  PGK1  PGK2  PGM1  BGN  DCN  PYGL  PYGM  PYGB  PGAM1  PGAM2  BPGM  PCK1  PCK2  PAPSS1  PRELP  PC  RAE1  RANBP2  RPIA  SLC26A1  SDC1  SDC2  SDC3  SDC4  ST3GAL1  ST3GAL2  ST3GAL4  ST3GAL3  SLC5A1  SLC5A2  SLC5A3  SLC5A4  SGSH  SI  TALDO1  TKT  TPR  TREH  SLC25A1  NUP155  RPS27A  UBA52  CHIA  CHIT1  NUP133  NUP54  NUPL1  RPE  TPI1  AGRN  DCXR  EPM2A  KIAA1199  NUP205  NHLRC1  NUP93  NUP98  BCAN  SLC45A3  STAB2  SEH1L  ST3GAL6  NUPL2  NUP85  NUP35  ADPGK  B3GNT4  B3GNT7  B3GALT6  CSGALNACT1  CSGALNACT2  CHPF2  CHSY1  CHPF  CHSY3  CHST1  CHST2  CHST3  CHST5  CHST6  CHST7  CHST9  CHST11  CHST12  CHST13  CHST14  CSPG4  CSPG5  DSEL  DSE  GLB1L  GLYCTK  GPC2  SLC2A14  HS6ST1  HS6ST2  HS6ST3  HGSNAT  HPSE2  HPSE  HS2ST1  HYAL1  HYAL3  LYVE1  NDST3  NDST4  NUP188  HS3ST1  HS3ST2  HS3ST3A1  HS3ST3B1  HS3ST4  HS3ST5  HS3ST6  PGM2  NUP210  PPP1R3C  RSC1A1  SLC35B2  SLC35B3  SLC35D2  SLC5A10  CHST15  UST  XYLB  XYLT1  XYLT2  G6PC2  G6PC3  SLC5A9  GNPDA2  POM121  POM121C  SLC37A4  PAPSS2  GCK  PPP2R5D  UGP2  UBC  UBB  SLC3A2  GOT1  GOT2  ACAD8  ACADSB  NDUFAB1  ASPA  AGXT2  GPT  ARG2  ARG1  ASL  ASNS  ASS1  SAT1  TAT  BCAT2  BCAT1  BCKDK  BHMT  BBOX1  CBS  CNDP2  CTH  CPS1  CRYM  CSAD  CDO1  HIBADH  ALDH7A1  AMD1  HDC  ODC1  DDC  ALDH9A1  GLUD1  GLUD2  QDPR  SLC25A10  DLD  DBH  SHFM1  FAH  IL4I1  FOLH1  FTCD  GAMT  GATM  GCDH  GCSH  GLDC  AMT  CGA  GNMT  GLUL  GLS  GLS2  SHMT1  GCLM  GCLC  HAO1  HSD17B10  HGD  HNMT  HPD  HAL  UROC1  IDO1  INMT  DIO3  IVD  GCAT  CKB  CKM  CKMT2  CKMT1A  KYNU  SLC7A5  LIAS  LIPT1  GSTZ1  SLC45A2  AIMP1  AIMP2  EEF1E1  MCCC1  MCCC2  MTR  MAT1A  ALDH6A1  MTAP  MTRR  NAALAD2  NNMT  NQO1  OAT  OAZ2  OAZ3  OAZ1  DBT  BCKDHA  BCKDHB  AZIN1  SLC25A21  OGDH  DLST  DLAT  PDHA1  PDHB  PDHX  SLC25A15  SLC25A2  OTC  DAO  DDO  PAH  PCBD1  PNMT  PAPSS1  PYCR1  PRODH  PSMC5  PSMA8  ALDH4A1  OCA2  RPS4Y2  SLC6A7  SLC6A8  SLC6A11  SLC6A12  AHCY  SECISBP2  EEFSEC  PHGDH  PSPH  PSAT1  SLC5A5  AANAT  PIPOX  AGMAT  SRM  SMS  AGXT  SQRDL  SUOX  DARS  EPRS  IARS  KARS  MARS  QARS  RARS  SARS  TDO2  SERINC3  SERINC1  ACAT1  TST  TMLHE  TXNRD1  TSHB  TYRP1  DCT  TYR  PSMD14  NAGS  HIBCH  AASS  AADAT  KMO  ACMSD  RPS10  RPS11  RPS12  RPS13  RPS14  RPS15  RPS16  RPS17  RPS18  RPS19  RPS15A  RPS2  RPS20  RPS21  RPS23  RPS24  RPS25  RPS26  RPS27  RPS27A  RPS28  RPS29  RPS3  FAU  RPS3A  RPS4X  RPS4Y1  RPS5  RPS6  RPS7  RPS8  RPS9  RPSA  RPLP0  RPLP1  RPLP2  RPL10  RPL10A  RPL11  RPL12  RPL13  RPL13A  RPL14  RPL15  RPL17  RPL18  RPL18A  RPL19  RPL21  RPL22  RPL23  RPL23A  RPL24  RPL26  RPL26L1  RPL27  RPL27A  RPL28  RPL29  RPL3  RPL3L  RPL30  RPL31  RPL32  RPL34  RPL35  RPL35A  RPL36  RPL37  RPL37A  RPL38  RPL39  RPL4  UBA52  RPL41  RPL36A  RPL5  RPL6  RPL7  RPL7A  RPL8  RPL9  AUH  PSMC1  PSMC4  PSMC2  PSMC3  PSMC6  PSMA1  PSMA2  PSMA3  PSMA4  PSMA5  PSMA6  PSMA7  PSMB1  PSMB2  PSMB3  PSMB4  PSMB5  PSMB6  PSMB7  PSMB8  PSMB9  PSMB10  PSMD1  PSMD2  PSMD3  PSMD4  PSMD5  PSMD6  PSMD7  PSMD8  PSMD9  PSMD10  PSMD11  PSMD12  PSMD13  PSME1  PSME2  PSME3  PSMF1  RPL39L  SERINC2  TPH2  PAOX  ADO  GRHPR  CCBL1  ALDH18A1  RPS27L  SMOX  LARS  AFMID  AMDHD1  APIP  ASRGL1  BHMT2  C9orf41  DHTKD1  DUOX1  DUOX2  MRI1  ENOPH1  ETHE1  GADL1  GSR  IDO2  CCBL2  ASPG  ADI1  NMRAL1  PYCR2  PYCRL  PPM1K  PSMB11  PSME4  PSTK  RPL10L  RPL22L1  RPL36AL  SCLY  SEPSECS  CARNS1  LIPT2  SERINC4  SERINC5  PAPSS2  TH  TPO  TPH1  ASMT  DIO1  IYD  GPT2  DIO2  ADC  PPP2R1A  PPP2R1B  ADRA2A  PRKAA2  PRKAB2  PRKAG2  ACLY  CHRM3  SLC25A4  SLC25A5  SLC25A6  ADIPOQ  ARL2  CACNA1A  CACNA1E  CACNB2  CACNB3  ACACB  ADCY1  ADCY2  ADCY3  ADCY4  ADCY5  ADCY6  ADCY7  ADCY8  ADCY9  PFKFB1  FASN  GNA11  GNA14  GNA15  GNAS  GNB1  GNB2  GNB3  GNB4  GNGT1  GNG3  GNG4  GNG5  GNG7  GNG8  GNG10  GNG11  GNG12  GNG13  GNGT2  GNAI1  GNAI2  GNAQ  GCGR  GCG  FFAR1  SLC2A1  SLC2A2  INS  ITPR3  KCNJ11  PRKAR1A  PRKAR1B  PRKAR2A  PRKAR2B  PRKACA  PRKACB  PRKACG  PRKCA  PKLR  MARCKS  MLX  PPP2CA  PPP2CB  PLCB1  PLCB2  PLCB3  AGPAT1  RAP1A  AHCYL1  STK11  SYT5  TALDO1  TKT  VAMP2  MLXIPL  ADIPOR1  ADIPOR2  GNG2  ADRA2C  CACNA2D2  ARL2BP  CACNA1C  GLP1R  AKAP5  KCNS3  IQGAP1  KCNB1  KCNG2  GNB5  RAPGEF3  SNAP25  STXBP1  CACNA1D  ABCC8  ACACA  ITPR2  ITPR1  PPP2R5D  STX1A  RAPGEF4  KCNC2  NT5C2  NT5E  ADA  ADK  AGXT2  AMPD1  AMPD2  AMPD3  APRT  UPB1  CAT  CDA  DCK  DCTD  DGUOK  DPYD  DPYS  ENTPD1  ENTPD2  ENTPD3  ENTPD4  ENTPD5  ENTPD6  GLRX  GMPR  GMPR2  GPX1  GMPS  GDA  HPRT1  IMPDH1  IMPDH2  AK1  AK2  AK5  CMPK1  GUK1  TK2  DTYMK  NME1  NME2  NME4  NUDT5  PNP  PPAT  GART  PFAS  PAICS  ADSL  ATIC  ADSS  CAD  UMPS  DHODH  RRM1  RRM2  TXN  TXNRD1  TYMP  TYMS  UCK1  UCK2  UPP1  XDH  TK1  ITPA  AK7  NT5C  NT5M  ADSSL1  UPP2  NT5C1A  NT5C1B  DUT  NUDT13  POMP  NUDT1  ADAL  DDX31  ENTPD7  ENTPD8  GSR  LHPP  NUDT15  NUDT16  CTPS2  RRM2B  DCTPP1  NUDT18  NUDT9  NT5E  ABCD4  AOX1  AKR1B10  AKR1C1  AKR1C3  AKR1C4  APOA1  APOA2  APOA4  APOB  APOC2  APOC3  APOE  APOM  HLCS  BST1  BTD  MTHFD1  CD38  CTRC  ACACB  CLPS  CYP24A1  CYP27B1  CYP8B1  CTRB1  CYB5A  SLC25A19  DHFR  FASN  SLC19A1  FOLR2  ALDH1L1  MTHFS  SLC25A16  GPHN  SHMT1  SHMT2  GPC1  GPC3  GPC4  GPC5  GPC6  GSTO1  SLC2A1  SLC2A3  GIF  LDLR  LGMN  LPL  PNLIP  LRP1  LRP2  MCCC1  MCCC2  MTR  SLC25A32  MOCS3  ABCC1  MOCS2  MTHFD2  MTHFR  MTRR  MUT  QPRT  COASY  CYB5R3  NNMT  ENPP1  ENPP2  ENPP3  NAMPT  PCCA  PCCB  PDXK  HSPG2  PTGS2  PANK2  PANK3  PANK4  ACP5  NADK  PARP4  PTGIS  PC  RBP1  RBP2  RBP4  SDC1  SDC2  SDC3  SDC4  SLC5A6  LRP12  SLC23A1  SLC23A2  TCN1  TCN2  SLC19A2  PRSS1  PRSS3  TTR  VNN1  VNN2  GC  LRAT  AMN  PARP9  BCMO1  BCO2  GSTO2  MMAA  MMAB  NMNAT1  NMNAT2  PNPO  RDH11  RFK  THTPA  AGRN  LRP10  LRP8  NMNAT3  PPCS  TPK1  VKORC1  PPCDC  FLAD1  NADSYN1  FPGS  AASDHPPT  SLC19A3  SLC46A1  CYP2R1  APOA1BP  ALDH1L2  MMADHC  CD320  CTRB2  CUBN  DHFRL1  GPC2  GPIHBP1  LMBRD1  MMACHC  MOCOS  NFS1  NUDT12  PARP10  PARP14  PARP16  PARP6  PARP8  PDZD11  RFT1  PLB1  NAPRT1  SLC22A13  UBIAD1  VKORC1L1  MTHFD2L  SLC5A8  CARKD  MTHFD1L  ACACA  PANK1  MOCS1  ACO2  NDUFAB1  ATP5G1  ATP5G2  ATP5G3  ATP5A1  ATP5B  ATP5D  ATP5E  ATP5F1  ATP5C1  ATP5I  ATP5J2  ATP5L  ATP5O  ATP5H  ATP5J  ATP5S  BSG  SDHC  NDUFAF1  CS  COX7A2L  COX5A  COX5B  COX6A1  COX6B1  COX6C  COX7B  COX7C  COX8A  COX11  COX4I1  CYC1  CYCS  SDHA  SDHB  SDHD  DLD  ETFA  ETFB  FH  IDH3A  IDH3B  IDH3G  IDH2  LDHA  LDHB  LDHC  LDHAL6B  GLO1  LRPPRC  MDH2  SLC16A1  SLC16A8  SLC16A3  NDUFA7  NDUFC2  NDUFA12  NDUFB3  NDUFA6  NDUFB4  NDUFA13  NDUFB6  NDUFB7  NDUFB9  NDUFA2  NDUFA3  NDUFB8  NDUFB10  NDUFB2  NDUFC1  NDUFA1  NDUFB1  NDUFS5  NDUFB5  NNT  NDUFS1  NDUFV1  NDUFS2  NDUFA10  NDUFA9  NDUFA5  NDUFS3  NDUFV2  NDUFS8  NDUFS7  NDUFA4  NDUFS6  NDUFV3  NDUFA8  NDUFS4  OGDH  DLST  DLAT  PDHA1  PDHB  PDHX  PDK1  PDK2  PDK3  PDK4  PDP1  PDP2  PPARD  RXRA  SUCLA2  SUCLG2  SCO1  SCO2  SUCLG1  SURF1  TRAP1  UCP1  UCP2  SLC25A27  UQCRC1  UQCRC2  UQCRB  UQCRH  UQCRFS1  UQCRQ  UQCR10  UQCR11  ACAD9  NDUFAF4  NUBPL  NDUFB11  COX18  TACO1  LDHAL6A  NDUFA11  ECSIT  NDUFAF3  COX16  COX19  COQ10A  COQ10B  D2HGDH  ADHFE1  L2HGDH  NDUFAF2  TMEM126B  PDPR  SLC25A14  UCP3  ETFDH  HAGH |
