# Supplementary figures and images for: Identification of a Metabolic Reprogramming-Associated Risk Model Related to Prognosis, Immune Microenvironment, and Immunotherapy of Stomach Adenocarcinoma
Source: J Oncol. 2022 Sep 21;2022:7248572. doi: 10.1155/2022/7248572 (PMC9519326; doi:10.1155/2022/7248572)

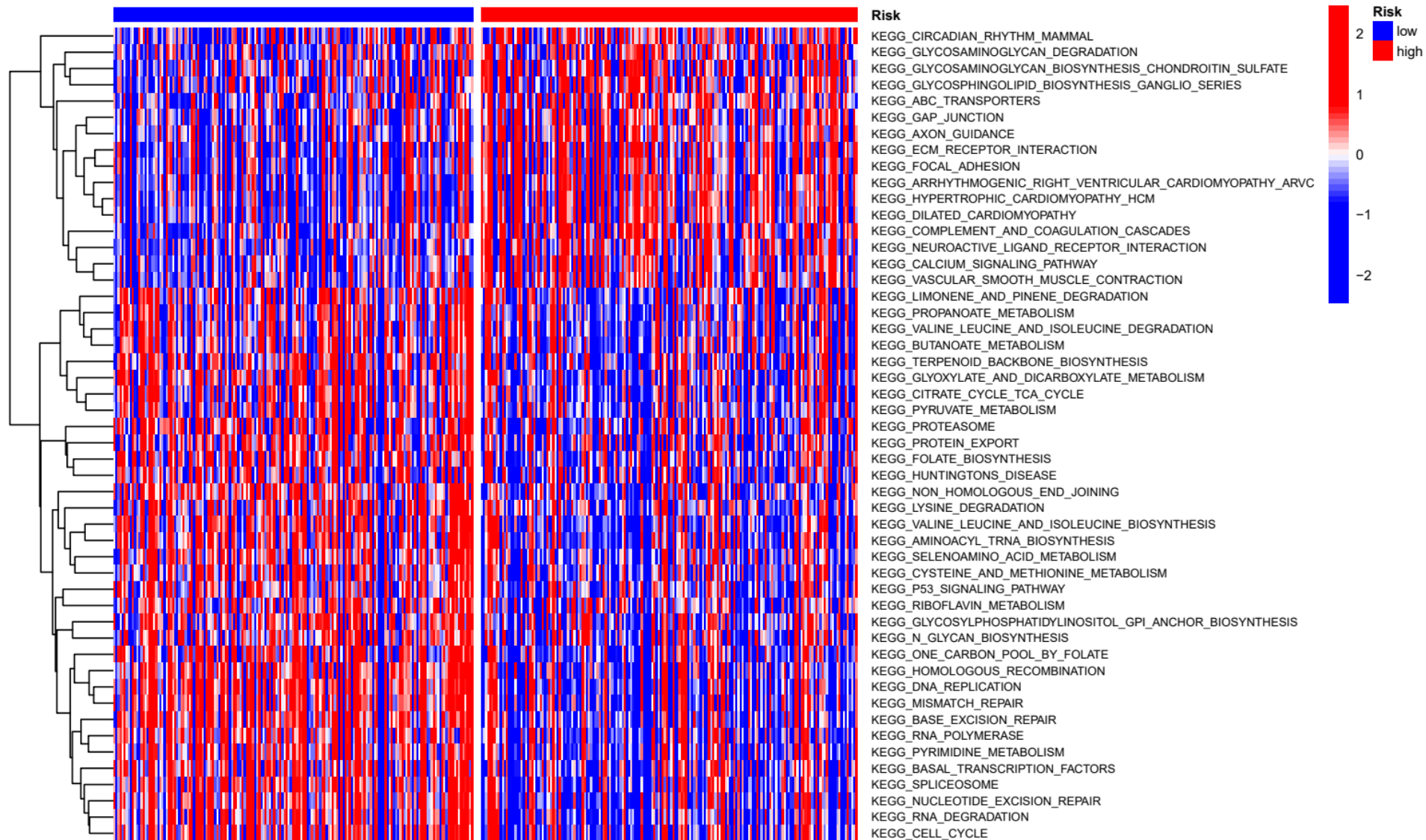

Supplement: Supplementary Materials — Figure S1. The GSVA enrichment heatmap between two risk score groups from the entire TCGA-STAD set. Table S1. The details of MRGs.() [file 7248572.f1.zip › supplementary Figure 1.pdf]
